# Effects of Zinc Deficiency and Supplementation on Hippocampal ZnT3, NOGO-A, IL-6, and SESN2 Expression and Oxidative Stress Markers in a Cuprizone-Induced Toxic Demyelination Model

**DOI:** 10.3390/ijms27209329

**Published:** 2026-10-20

**Authors:** Adem Atacak, Elif Gulbahce-Mutlu, Gozde Acar, Saltuk Bugra Baltaci, Rasim Mogulkoc, Abdulkerim Kasim Baltaci

**Affiliations:** 1 Department of Phsiology, Medical Faculty, Selçuk University, 42250 Konya, Türkiye; adematacak04@gmail.com (A.A.); gzdcr67@gmail.com (G.A.); mogulkoc@selcuk.edu.tr (R.M.); 2 Department of Medical Biology, Medical Faculty, KTO Karatay University, 42020 Konya, Türkiye; elifgulbahcemutlu@gmail.com; 3 Department of Phsiology, Medical Faculty, Istanbul Medipol University, 34810 Istanbul, Türkiye; bugrabaltaci@icloud.com

**Keywords:** toxic demyelination model, zinc, hippocampus, ZnT3, NOGO-A, IL-6, SESN2, oxidative stress

## Abstract

This study investigated the effects of zinc deficiency and supplementation on oxidative stress markers and the expression of ZnT3, NOGO-A, IL-6, and SESN2 in hippocampal tissue within a cuprizone (CPZ)-induced toxic demyelination model. Methods: Forty-six adult male Wistar rats were divided into five groups: Control (*n* = 6), Sham (*n* = 10), CPZ (*n* = 10), CPZ + zinc-deficient diet (*n* = 10), and CPZ + zinc supplementation (5 mg/kg/day, i.p., *n* = 10). Toxic demyelination was induced by continuous cuprizone administration for eight weeks. At the end of the experimental period, hippocampal malondialdehyde (MDA) and glutathione (GSH) levels were measured; additionally, hippocampal ZnT3, NOGO-A, IL-6, and SESN2 mRNA expression levels were analyzed using quantitative real-time PCR. Cuprizone administration led to significant hippocampal oxidative stress, characterized by increased MDA levels and decreased GSH concentrations (*p* < 0.05); these oxidative changes were further exacerbated in CPZ-treated animals fed a zinc-deficient diet (*p* < 0.05). Furthermore, CPZ-induced demyelination suppressed ZnT3 expression in hippocampal tissue while significantly increasing NOGO-A, IL-6, and SESN2 mRNA expression (*p* < 0.05). These molecular alterations were more pronounced in the zinc-deficient CPZ group (*p* < 0.05). Intraperitoneal zinc supplementation effectively mitigated these changes by suppressing oxidative stress, attenuating the upregulation of NOGO-A, IL-6, and SESN2 transcripts, and restoring ZnT3 expression to levels comparable to controls (*p* < 0.05). While dietary zinc deficiency exacerbated hippocampal oxidative stress, neuroinflammatory transcripts, and molecular markers associated with neurodegeneration in the cuprizone model, zinc supplementation restored oxidative balance; increased ZnT3 expression; and suppressed the upregulation of NOGO-A, IL-6, and SESN2. These findings suggest that zinc homeostasis significantly modulates cuprizone-induced hippocampal damage and that zinc supplementation may exert protective transcriptional and antioxidant effects during toxic demyelination.

## 1. Introduction

Multiple sclerosis (MS) is a chronic disease characterized by demyelination, neuroinflammation, and progressive neurodegeneration in the central nervous system [1,2]. During the disease process, oligodendrocyte loss, myelin destruction, and axonal damage occur, while the accompanying inflammatory and oxidative mechanisms cause progressive deterioration of neurological functions [1,2]. In addition to motor function losses, cognitive impairments are among the important clinical features of MS, and learning and memory processes, especially those related to the hippocampus, are negatively affected by the disease [2,3]. One of the most common models used to investigate these pathological processes is the toxic demyelination model induced by cuprizone. Cuprizone application causes oligodendrocyte loss and demyelination in certain brain regions, while also allowing the evaluation of subsequent remyelination processes [4]. The fact that the hippocampus is one of the regions sensitive to cuprizone makes the examination of molecular changes in this region critical [5].

In recent years, interest in the role of trace elements—and zinc in particular—in the cuprizone-induced toxic demyelination model has grown [6]. Zinc is an essential trace element involved in synaptic transmission, neuroplasticity, immune response, and antioxidant defense mechanisms. Disruption of zinc homeostasis in brain tissue is associated with the development of neurodegenerative diseases [7]. Zinc transporter protein 3 (ZnT3), which is responsible for zinc transport into synaptic vesicles, is found in high concentrations—particularly in the hippocampus—and plays a critical role in learning, memory, and neurogenesis. Changes in ZnT3 levels have been reported to be associated with synaptic dysfunction and cognitive impairment [8].

NOGO-A, interleukin-6 (IL-6), and sestrin-2 (SESN2) are among the other key molecules influencing neuroinflammation and neurodegeneration in the cuprizone-induced toxic demyelination model [9,10,11]. NOGO-A is a myelin-derived inhibitory protein that suppresses axonal growth and synaptic remodeling; it has been associated with the loss of neuroplasticity and insufficient remyelination in the toxic demyelination model [10]. IL-6 is a significant cytokine that exhibits pro-inflammatory properties and shows increased expression in the toxic demyelination model [9]. In contrast, SESN2 is a stress-response protein that exerts protective effects against oxidative stress and cellular damage, playing a role in the regulation of antioxidant defense and autophagic processes [11]. A combined assessment of changes in these molecules within hippocampal tissue may contribute to a better understanding of the neurodegenerative processes associated with the toxic demyelination model.

It is known that oxidative stress plays a critical role in disease progression within the cuprizone-induced toxic demyelination model [9,12]. In both experimental and clinical studies, elevated levels of malondialdehyde (MDA)—a key indicator of lipid peroxidation—and reduced levels of glutathione (GSH)—a major endogenous antioxidant—are recognized as biochemical markers of neurodegeneration [12]. Therefore, assessing MDA and GSH levels is important for elucidating the molecular aspects of hippocampal tissue damage.

However, there are no studies in the literature evaluating the combined effects of zinc deficiency and supplementation on hippocampal ZnT3, NOGO-A, IL-6, and SESN2 expression in the cuprizone-induced toxic demyelination model. Accordingly, this study was designed as a preliminary investigation—aimed at exploring the effects of zinc status on hippocampal oxidative stress and molecular markers within a cuprizone-induced toxic demyelination model—to provide foundational data for future research.

## 2. Results

### 2.1. MDA and GSH Results in Hippocampus Tissue

**Figure 1:**
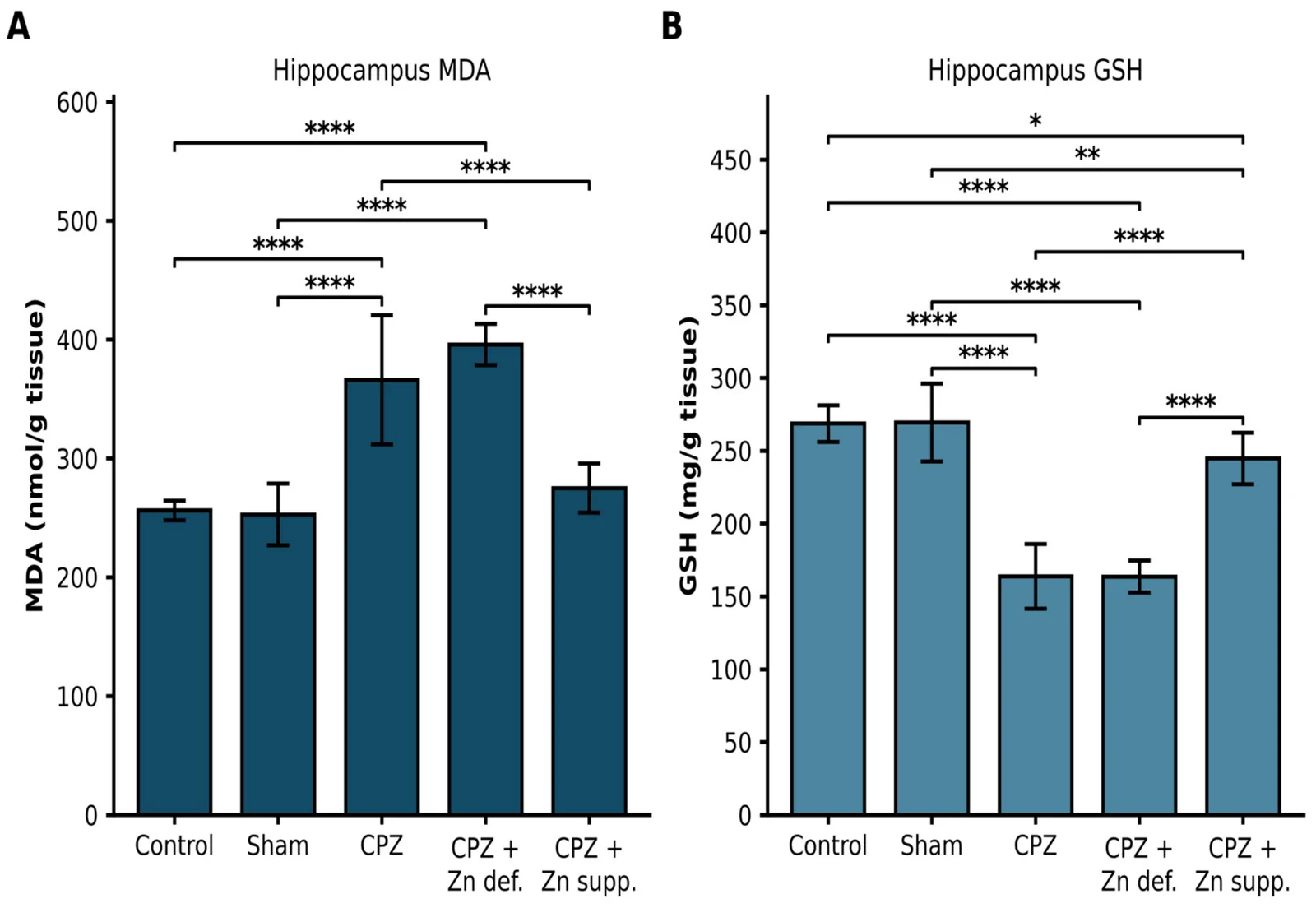
Effects of cuprizone-induced toxic demyelination and zinc status on hippocampal MDA (**A**) and GSH (**B**) levels. Data are expressed as mean ± standard deviation (SD) (*n* = 6 for Control; *n* = 10 for all other groups). Brackets indicate statistically significant pairwise differences between groups: * *p* < 0.05, ** *p* < 0.01, **** *p* < 0.0001. Groups not connected by a bracket did not differ significantly. Abbreviations: MDA, malondialdehyde; GSH, reduced glutathione; CPZ, Cuprizone-induced toxic demyelination; Zn, zinc; def., deficient; supp., supplementation.

In our study, the highest MDA and lowest GSH levels in hippocampal tissue were obtained in CPZ group (Group 3) and CPZ group fed a zinc-deficient diet (Group 4) (*p* < 0.05). Compared to the CPZ groups without zinc supplementation (Groups 3 and 4), zinc supplementation significantly reduced hippocampal MDA values (*p* < 0.05) and significantly increased GSH values (*p* < 0.05) in the CPZ (Group 5) group (Table 1 and Figure 1).

### 2.2. Hippocampus NOGO-A and ZnT3 Gene Expression Results

**Figure 2:**
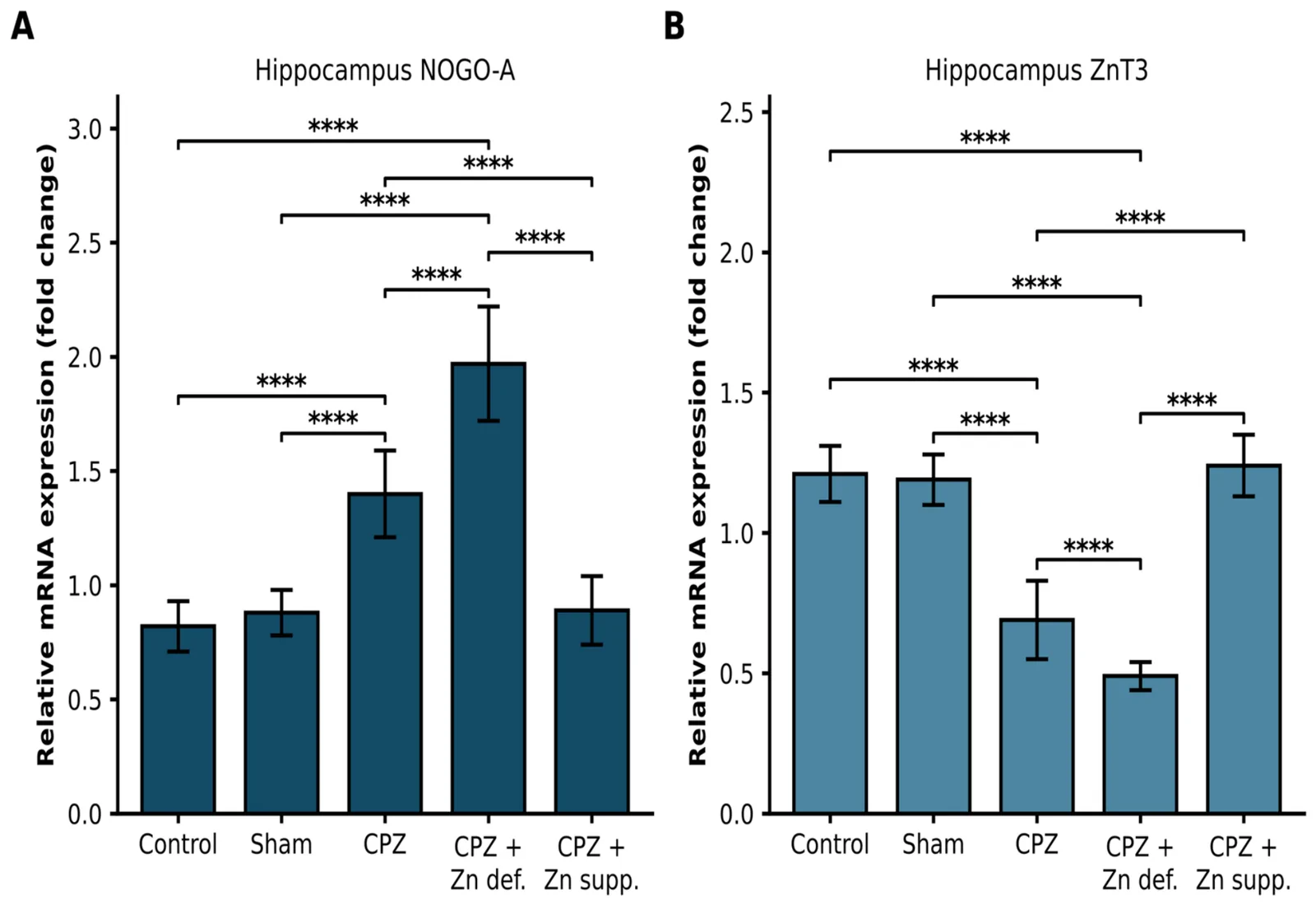
Relative mRNA expression levels of hippocampal NOGO-A (**A**) and ZnT3 (**B**) across experimental groups. Data are presented as mean relative fold change ± standard deviation (SD) calculated using the 2^−ΔΔCt^ method (*n* = 6 for Control; *n* = 10 for all other groups). Brackets indicate statistically significant pairwise differences between groups: **** *p* < 0.0001. Groups not connected by a bracket did not differ significantly. Abbreviations: NOGO-A, neurite outgrowth inhibitor A; ZnT3, zinc transporter 3; CPZ, Cuprizone-induced toxic demyelination; Zn, zinc; def., deficient; supp., supplementation.

The highest NOGO-A gene expression in hippocampus tissue was obtained in the CPZ group fed a zinc-deficient diet (Group 4) (*p* < 0.05). In the CPZ group (Group 3) fed with standard rat diet, NOGO-A gene expression in hippocampal tissue was lower than in Group 4 (*p* < 0.05), but significantly higher than all other groups (*p* < 0.05). Compared to the unsupported groups, zinc supplementation pre-vented the increase in hippocampal NOGO-A gene expression levels in the CPZ group (Group 5), bringing them back to control values (*p* < 0.05) (Table 2 and Figure 2).

The lowest ZnT3 gene expression in hippocampal tissue was obtained in the CPZ group (Group 4) fed with a zinc-deficient diet (*p* < 0.05). In the CPZ group (Group 3) fed with standard rat diet, ZnT3 gene expression in hippocampal tissue was higher than in Group 4 (*p* < 0.05), but significantly lower than all other groups (*p* < 0.05). Compared to the unsupported groups, zinc supplementation increased hippocampal ZnT3 gene expression levels in the CPZ group (Group 5), bringing them back to control values (*p* < 0.05; Table 2 and Figure 2).

### 2.3. Hippocampus IL-6 and SESN2 Gene Expression Results

**Figure 3:**
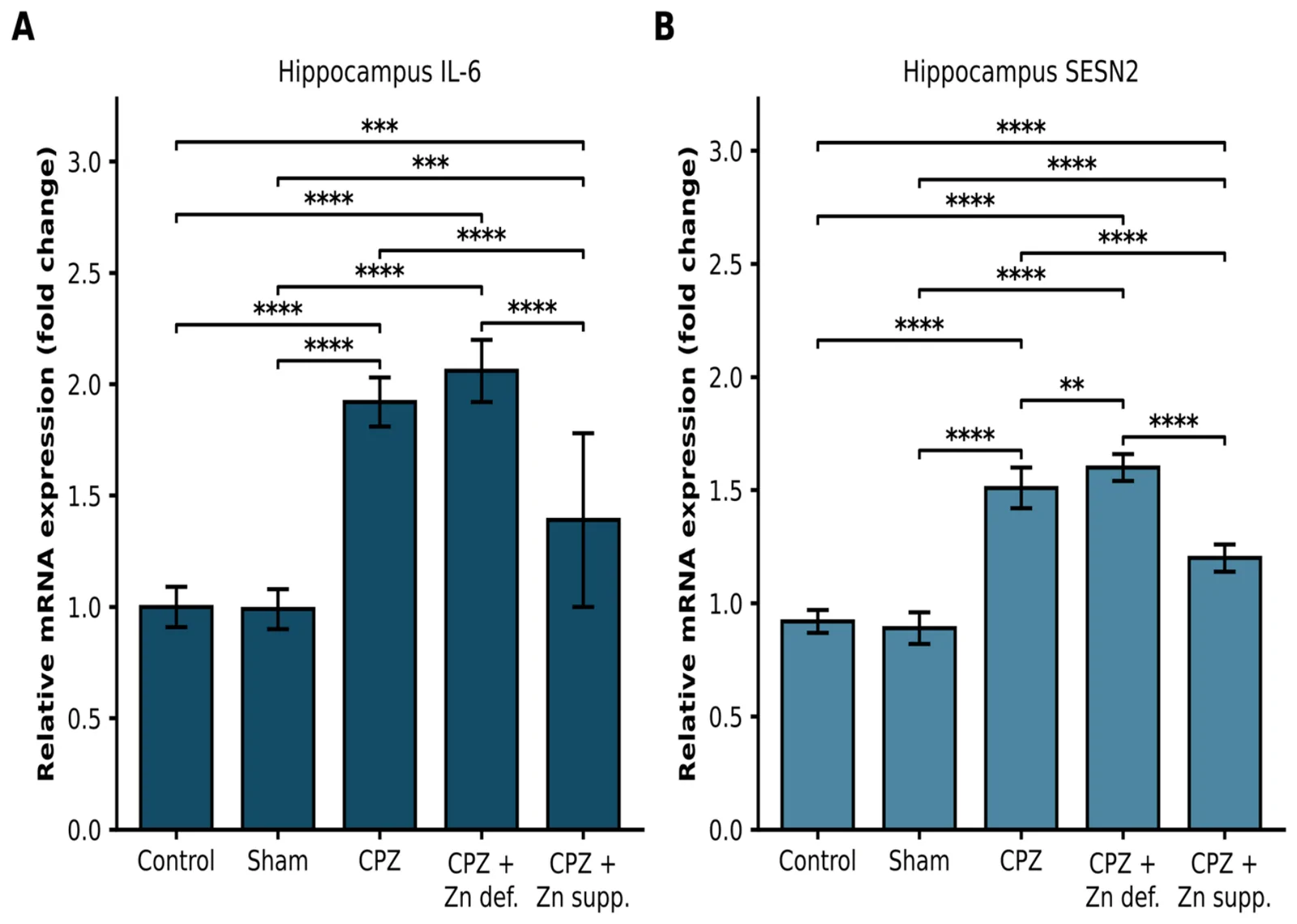
Relative mRNA expression levels of hippocampal IL-6 (**A**) and SESN2 (**B**) across experimental groups. Data are presented as mean relative fold change ± standard deviation (SD) calculated using the 2^−ΔΔCt^ method (*n* = 6 for Control; *n* = 10 for all other groups). Brackets indicate statistically significant pairwise differences between groups: ** *p* < 0.01, *** *p* < 0.001, **** *p* < 0.0001. Groups not connected by a bracket did not differ significantly. Abbreviations: IL-6, interleukin 6; SESN2, sestrin 2; CPZ, Cuprizone-induced toxic demyelination; Zn, zinc; def., deficient; supp., supplementation.

In our study, we obtained the highest IL-6 gene expression levels in hippocampus tissue in the CPZ (Group 3) and CPZ (Group 4) groups fed a zinc-deficient diet (*p* < 0.05). Compared to the CPZ groups without zinc supplementation (Groups 3 and 4), zinc supplementation significantly reduced hippocampus IL-6 gene expression values in the CPZ (Group 5) group (*p* < 0.05; Table 3 and Figure 3).

The highest SESN2 gene expression values in hippocampus tissue were obtained in the CPZ (Group 4) group fed a zinc-deficient diet (*p* < 0.05). The SESN2 gene expression in the hippocampus tissue of the CPZ group (Group 3) fed a standard rat diet was lower than Group 4 (*p* < 0.05), but significantly higher than all other groups (*p* < 0.05). Compared to CPZ groups without zinc supplementation (Groups 3 and 4), zinc supplementation significantly reduced hippocampal SESN2 gene expression in CPZ group (Group 5) (*p* < 0.05; Table 3 and Figure 3).

## 3. Discussion

Toxic demyelination in the central nervous system (CNS) is primarily characterized by progressive myelin breakdown, oligodendrocyte loss, and axonal damage—pathological processes that lead to structural and functional impairments [13,14]. The cuprizone model is widely used in experimental studies to investigate the cellular and molecular mechanisms underlying toxic demyelination [3,13,14,15]. The hippocampus, which plays a critical role in memory consolidation and retrieval, is one of the regions frequently affected by toxic demyelination; demyelinating damage in this area often manifests as significant cognitive deficits [15,16].

In our study, we evaluated molecular markers associated with hippocampal oxidative stress, inflammation, and neuroplasticity (MDA, GSH, ZnT3, NOGO-A, IL-6, SESN2) in rats subjected to a cuprizone-induced experimental toxic demyelination model; additionally, we investigated the effects of dietary zinc levels on these parameters.

### 3.1. Oxidative Stress and Antioxidant Defense: MDA and GSH

The highest malondialdehyde (MDA) and lowest glutathione (GSH) levels were found in the CPZ (Group 3) and CPZ fed a zinc-deficient diet (Group 4) groups. In contrast, in the CPZ group receiving intraperitoneal zinc supplementation (Group 5), hippocampal MDA levels were significantly decreased, while GSH levels were significantly increased compared to the groups without supplementation. Cuprizon is a neurotoxic copper chelator that triggers demyelination and allows evaluation of therapeutic effects on remyelination [17]. Since the CPZ-induced toxic demyelination model is a process characterized by microglial activation and oligodendrocyte loss, an increase in reactive oxygen species (ROS) and associated tissue damage is observed during this process [18,19]. In our study, the observation of hippocampal MDA increase and GSH suppression in the CPZ group fed a standard diet (Group 3) is consistent with studies in the literature reporting cuprizone-induced oxidative stress and hippocampal damage [15,19]. Zinc is a potent antioxidant element, and its deficiency may play a role in the development of neurodegenerative diseases and the pathogenesis of demyelination through oxidative stress [20,21]. A clinical study has reported that zinc levels in the body fluids of subjects with demyelination-associated neurodegeneration are lower compared to healthy individuals and that this may be associated with disease severity [22]. Although the MDA levels of the CPZ group fed a zinc-deficient diet (Group 4) were numerically higher than those of the CPZ group fed a standard rat diet (Group 3) in our study, no statistically significant difference was found; this may be due to individual variations or the duration of the diet. However, the significant suppression of MDA levels and the increase in GSH levels in the CPZ group receiving zinc supplementation (Group 5) indicate that zinc strengthens antioxidant capacity and reduces hippocampal damage.

### 3.2. Zinc Homeostasis and Synaptic Function: ZnT3

Zinc plays a critical role in fundamental biological processes such as enzymatic reactions, cell division, differentiation, and synaptic transmission; zinc transporter proteins are vital in regulating these processes [23,24]. ZnT3, a key zinc transporter, is one of the most abundant membrane proteins in synaptic vesicles in the brain [25]. Although its physiological functions in the central nervous system have been better defined recently, the association of ZnT3 with memory performance is clear [8,24]. Genetic deletion of ZnT3 and the resulting decrease in synaptic zinc concentration have been reported to be associated with age-aggravating memory impairments [8,24].

A decline in cognitive performance and memory loss are commonly reported in neurodegenerative diseases [26]. These impairments have been associated with specific damage, such as demyelinating lesions and/or hippocampal atrophy [27]. Hippocampal atrophy and demyelination—processes affecting the formation and maintenance of long-term memory—are frequently observed in neurodegenerative diseases [28]; consequently, it is recognized that cognitive impairment, particularly in the context of demyelination, may also be linked to hippocampal damage [29].

Interest in the relationship between hippocampal zinc regulation and cognitive impairments has increased in recent years. It has been shown that impaired cognitive performance and decreased hippocampal ZnT3 levels in an experimental Alzheimer’s model can be partially reversed with zinc supplementation [8]. It has been reported that hippocampal zinc, reduced by deletion of the ZnT3 gene, leads to cognitive impairment in 6-month-old mice [30]. Similarly, it has been suggested that zinc supplementation im-proves memory performance in rats and that zinc could be used as a potential molecule in the treatment of memory disorders in light of these findings [31]. However, there is no study directly examining the relationship between zinc, cognitive performance, and the hippocampus in the CPZ-induced toxic demyelination model. In our study, the lowest hippocampal ZnT3 gene expression was detected in the CPZ group fed a zinc-deficient diet (Group 4). This finding is consistent with data reported by Adlard et al. [30], indicating that hippocampal zinc regulation is impaired by ZnT3 gene deletion. Furthermore, it is noteworthy that hippocampal ZnT3 gene expression was also reduced in the CPZ group fed a standard diet. Many researchers have reported that toxic demyelination leads to impaired hippocampal function and subsequent cognitive impairment [26,28,29]. In addition to these findings, our study demonstrates that the suppression of ZnT3 gene expression may play a critical role in the impaired hippocampal function observed in the CPZ-induced toxic demyelination model.

On the other hand, in the CPZ group receiving zinc supplementation (Group 5), hippocampal ZnT3 gene expression levels were significantly increased and approached control values compared to the groups without supplementation. The finding that decreased hippocampal ZnT3 expression in an experimental Alzheimer’s model can be restored with zinc supplementation [8] is consistent with the increase in ZnT3 observed following zinc supplementation in the CPZ-induced toxic demyelination model used in our study.

### 3.3. Axonal Inhibition and Neuroplasticity: NOGO-A

Toxic demyelination is a critical neuropathological process affecting the central nervous system (CNS), characterized by inflammatory cascades, oligodendrocyte injury, and severe axonal damage [10]. NOGO-A is a 1192-amino acid myelin glycoprotein localized in the CNS and is among the myelin-derived inhibitory factors that inhibit axonal growth [32]. Molecular studies have shown that NOGO-A is expressed in oligodendrocytes, motor neurons, and interneurons in the spinal cord, as well as in sensory neurons in the dorsal root ganglia; and also in the cortex, hippocampus, and cerebellum [32,33].

A significant regulatory function of NOGO-A on synaptic plasticity in the adult CNS has been identified in recent years [34]. NOGO-A, which plays a role in synaptic plasticity and memory formation, also plays a critical role in the remodeling of neural circuits [35]; therefore, it is expected to be effective in hippocampal functions [34,36]. Due to its role in neuro-regeneration, NOGO-A contributes to pathogenic processes in a wide variety of neurological diseases [34,36]. In particular, NOGO-A levels, which are a potent inhibitor of neurite growth, have been reported to increase during MS [37]. In light of these findings, NOGO-A and its signaling receptors represent promising molecular targets for therapeutic interventions aimed at promoting axonal regeneration and tissue repair in toxic demyelination and related neurodegenerative disorders [35].

In our study, the highest hippocampal NOGO-A gene expression was detected in the CPZ group fed a zinc-deficient diet (Group 4). In the CPZ group fed a standard diet (Group 3), NOGO-A expression was lower than in Group 4, but significantly higher compared to all other groups. In the CPZ group receiving zinc supplementation (Group 5), NOGO-A gene expression levels were significantly suppressed and approached control values compared to the groups without supplementation. Our literature review did not reveal any studies directly examining the relationship between dietary zinc status and hippocampal NOGO-A levels within the context of a CPZ-induced toxic demyelination model. However, a study investigating the effect of dietary zinc status on hippocampal NOGO-A in an experimental Alzheimer’s model reported that zinc supplementation suppressed the elevated NOGO-A expression observed in both the Alzheimer’s and zinc-deficient Alzheimer’s groups [38]. This finding strongly parallels the suppression of the NOGO-A increase—observed in the CPZ and zinc-deficient CPZ groups in our study—following zinc supplementation. The present study is significant as it is the first to investigate the relationship between dietary zinc status and the hippocampus within a CPZ-induced toxic demyelination model.

### 3.4. Inflammation: IL-6

Cytokines are peptide molecules that exert anti- or pro-inflammatory effects and are actively involved in various cellular functions [39]. Pro-inflammatory cytokines can exacerbate existing structural damage by inducing harmful cascades under pathological conditions [40]. Pro-inflammatory cytokines, including IL-6, play a crucial role in the pathogenesis of neuroinflammation and demyelination by amplifying inflammatory responses in the central nervous system (CNS) [41].

IL-6, produced by immune cells including T cells, B cells, and macrophages/microglia, stimulates cellular responses to tissue injury; it further promotes the production of pro-inflammatory mediators upon binding to its receptor [42]. Fluctuations in IL-6 levels in the cerebrospinal fluid (CSF) have been significantly correlated with the severity of demyelinating lesions and clinical/radiological disease progression [43]. Although IL-6 has not yet been widely established as a direct single target in demyelinating conditions, it is suggested that the efficacy of various neuroprotective and immunomodulatory interventions may be partly attributed to their capacity to suppress pro-inflammatory cytokines such as IL-6 [41]. Increased hippocampal IL-6 expression has been consistently documented in demyelinating conditions [15]. Furthermore, elevated IL-6 levels represent a hallmark of progressive central demyelination, indicating that IL-6 suppression may serve as a potential therapeutic strategy in toxic demyelination [44]. Neurodegenerative disorders are frequently accompanied by high levels of central and circulating inflammatory markers such as IL-6 [45]. Elevated IL-6 in the CSF has also been linked to reduced neuroplasticity capacity during demyelinating pathology [43]. As an inflammatory cytokine, sustained elevation of IL-6 can exert direct neurotoxic effects on cerebral tissues [46].

In our study, the highest hippocampal IL-6 gene expression levels were detected in the CPZ group fed a standard diet (Group 3) and the CPZ group fed a zinc-deficient diet (Group 4). The upregulation of a key pro-inflammatory cytokine such as IL-6 in toxic demyelination is an expected outcome and aligns closely with the literature data summarized above. Notably, IL-6 levels were further elevated in the CPZ group subjected to dietary zinc deficiency. To date, no study has directly examined the complex interplay among toxic demyelination, neuroinflammation, and dietary zinc status. However, previous reports demonstrate that zinc deficiency leads to elevated IL-6 levels in peripheral tissues, whereas zinc supplementation prevents tissue injury by suppressing IL-6 expression [47]. These observations confirm that zinc homeostasis plays an integral role in IL-6 regulation, which parallels the exacerbated IL-6 expression observed in our zinc-deficient CPZ group. Importantly, in our study, hippocampal IL-6 gene expression levels were significantly reduced in the CPZ group receiving zinc supplementation (Group 5) compared to the unsupplemented CPZ groups (Groups 3 and 4). Exogenous administration of zinc–metallothionein-II (Zn-MT-II) has been shown to reduce neuroinflammation by suppressing IL-6 in experimental demyelination models [48]. Similarly, increased IL-6 levels in other chronic inflammatory models have been effectively attenuated by zinc supplementation [49], serving as a striking example of the zinc–IL-6 regulatory axis. In conclusion, elevated hippocampal IL-6 gene expression in both the cuprizone-induced toxic demyelination model and the zinc-deficient model was significantly suppressed following zinc supplementation. These findings suggest that zinc supplementation may represent a promising supportive approach to attenuate elevated IL-6 gene expression in toxic demyelination. This study is particularly significant as it is the first to directly elucidate the toxic demyelination–hippocampus–IL-6–zinc relationship.

### 3.5. Cellular Stress Response: SESN2

SESN2 is a stress-inducible metabolic regulator with antioxidant activity, acting as a key protein that protects cells against oxidative stress [50]. SESN2 gene expression increases significantly during inflammatory responses to provide protection against lipid peroxidation and prevent tissue damage [51]. It has also been reported that SESN2 expression is elevated in neurodegenerative diseases such as Alzheimer’s and Parkinson’s [52,53].

Our literature review did not identify any studies directly investigating the relationship between SESN2 and zinc in a CPZ-induced toxic demyelination model. However, it has been reported that the co-administration of chito oligosaccharides and zinc increases SESN2 expression and improves impaired ovarian function in mice with premature ovarian failure [54]. This finding contrasts with the increase in SESN2 observed in the CPZ group fed a zinc-deficient diet in our study; this discrepancy may stem from differences in tissue type, the specific experimental model system, and the duration or nature of the administered agents.

In the CPZ group fed a standard diet (Group 3), hippocampal SESN2 expression was lower than in Group 4 but significantly higher compared to all other groups. In a clinical study examining the relationship between demyelinating pathology and SESN2, it was reported that serum SESN2 levels in patients with neurodegenerative disease (average disease duration of 7.5 years) were significantly lower than in healthy controls; in light of this finding, researchers suggested that reduced SESN2 levels could serve as a potential biomarker for chronic neurodegenerative diseases [11]. The increased hippocampal SESN2 expression observed in our study’s rats subjected to CPZ-induced toxic demyelination (Group 3) appears to contrast with these clinical data. However, while the clinical study in question involved patients in the chronic stage of the disease who were undergoing long-term pharmacological treatment, SESN2 measurements in our study were performed at the conclusion of an 8-week cuprizone-induced toxic demyelination model. Our findings demonstrate that hippocampal SESN2 gene expression increases significantly in the acute toxic demyelination model and that this upregulation is further exacerbated under conditions of dietary zinc deficiency. This indicates a significant interplay between dietary zinc status and SESN2 gene expression during toxic demyelination.

Furthermore, hippocampal SESN2 expression in the toxic demyelination group receiving zinc supplementation was significantly suppressed compared to the un supplemented groups. This finding suggests that zinc supplementation may exert a regulatory and protective effect on hippocampal SESN2 expression. This research is particularly significant as it represents the first study to elucidate the relationship between dietary zinc levels and hippocampal SESN2 gene expression within a cuprizone-induced toxic demyelination model, indicating that this regulatory axis warrants further, more detailed investigation in future studies.

### 3.6. General Evaluation and Conclusion

In a cuprizone-induced toxic demyelination model, dietary zinc deficiency aggravated, whereas zinc supplementation significantly attenuated, hippocampal oxidative stress, disrupted zinc homeostasis, neuroinflammation, and stress-related molecular transcript alterations (ZnT3, NOGO-A, IL-6, and SESN2). These findings suggest that maintaining zinc homeostasis and implementing zinc supplementation may represent a promising protective and supportive approach against toxic demyelination-induced hippocampal damage.

## 4. Materials and Methods

### 4.1. Experimental Animals and Study Design

This study was conducted at Selçuk University Experimental Medicine Application and Research Center (SUDAM). All experimental procedures were approved by the SUDAM experimental animal ethics committee with decision number 2022-23 dated and 24 June 2022.

Animal Information: 46 adult male Wistar rats were used in the study. Adult male Wistar rats, aged 10–12 weeks and weighing 220 ± 20 g at the start of the study, were used.

Housing and Acclimatization: The rats were housed in standard polypropylene cages under controlled environmental conditions (temperature: 22 ± 2 °C; humidity: 55 ± 5%; 12 h light/dark cycle) following a 2-day acclimatization period prior to the start of experiments. Wood chip bedding was replaced every 2 days.

Humane Endpoints: Animals were monitored daily for signs of severe distress, excessive weight loss (>20% of initial body weight), lethargy, or severe motor impairment. (No animals met the humane endpoint criteria during the 8-week protocol).

The animals were divided into a total of 5 groups.

Control (*n* = 6)
Sham (*n* = 10)
CPZ (*n* = 10)
CPZ + Zinc Deficiency Diet (*n* = 10)
CPZ + Zinc Supplement (*n* = 10)

Randomization and Blinding: Following a 2-day acclimatization period, animals were randomly assigned to the experimental groups after being precisely weighed to ensure an equal distribution of baseline body weight across all groups. To minimize bias, blinding was implemented during molecular and biochemical evaluations. Tissue homogenates and cDNA samples were coded with anonymous identifiers by an independent researcher who was not involved in the laboratory analyses. Outcome assessors performing the biochemical assays (malondialdehyde [MDA] and reduced glutathione [GSH]) and quantitative real-time PCR (qRT-PCR) measurements remained fully blinded to group allocations until all primary statistical analyses were completed.

Determination of Animal Group Sizes: The decisions of the animal experimentation ethics committee were taken into account when determining the number of animals. The sample size of X animals per group was established based on standards established in similar experimental rodent models published in the peer-reviewed literature. This number ensures compliance with institutional Animal Ethics Committee guidelines regarding the minimum number of animals required for statistical reliability, while also providing sufficient statistical power to observe significant physiological changes in biomarker endpoints. When forming the study groups, a simple randomization method was used to assign the experimental animals to control and treatment groups, ensuring that each animal had an equal and independent chance of being assigned to any given group.

In the zinc-deficient diet groups, experimental feed with a zinc content of <50 µg/kg (Arden Research & Experiment, Ankara, Turkey) was used.

Serum zinc levels were determined in blood samples collected from the animals to verify the efficacy of the zinc treatments. The results showed that the highest serum zinc level was observed in the CPZ + Zn-supplemented group (Group 5), which was significantly elevated compared to all other experimental groups (*p* < 0.05). Conversely, the lowest level was found in the CPZ + zinc-deficient group (Group 4; *p* < 0.05 vs. all groups). Furthermore, serum zinc levels in the standard CPZ group (Group 3) were significantly higher than those in Group 4 (*p* < 0.0), yet significantly lower than those observed in the remaining groups (*p* < 0.05). These results demonstrate the validity of the zinc administration (Table 4).

### 4.2. Establishment of the Cuprizone-Induced Toxic Demyelination Model

Cuprizon [Bis(cyclohexanone) oxaldihydrazone, Sigma-Aldrich St. Louis-USA] was used to create an experimental demyelination model. Cuprizon was suspended in 1% carboxymethyl cellulose (CPZ) and administered orally via gavage for 8 weeks. The daily cuprizon dose was calculated as approximately 0.66 g/kg/day based on the average feed consumption of the animals. The sham group was administered only 1% CPZ for the same period [3].

Animals in the CPZ + Zinc Deficiency Diet group were fed zinc-deficient feed throughout the experiment, while the CPZ + Zinc Supplement group was administered zinc sulfate (5 mg/kg/day, i.p.) for 8 weeks [3]. The concept of administering zinc via the intraperitoneal route stemmed from two main reasons. The first was to ensure precise, daily, and reproducible systemic dosing per animal; the second was to eliminate individual variations in gastrointestinal zinc absorption and food intake that might arise during cuprizone administration.

**Figure 4:**
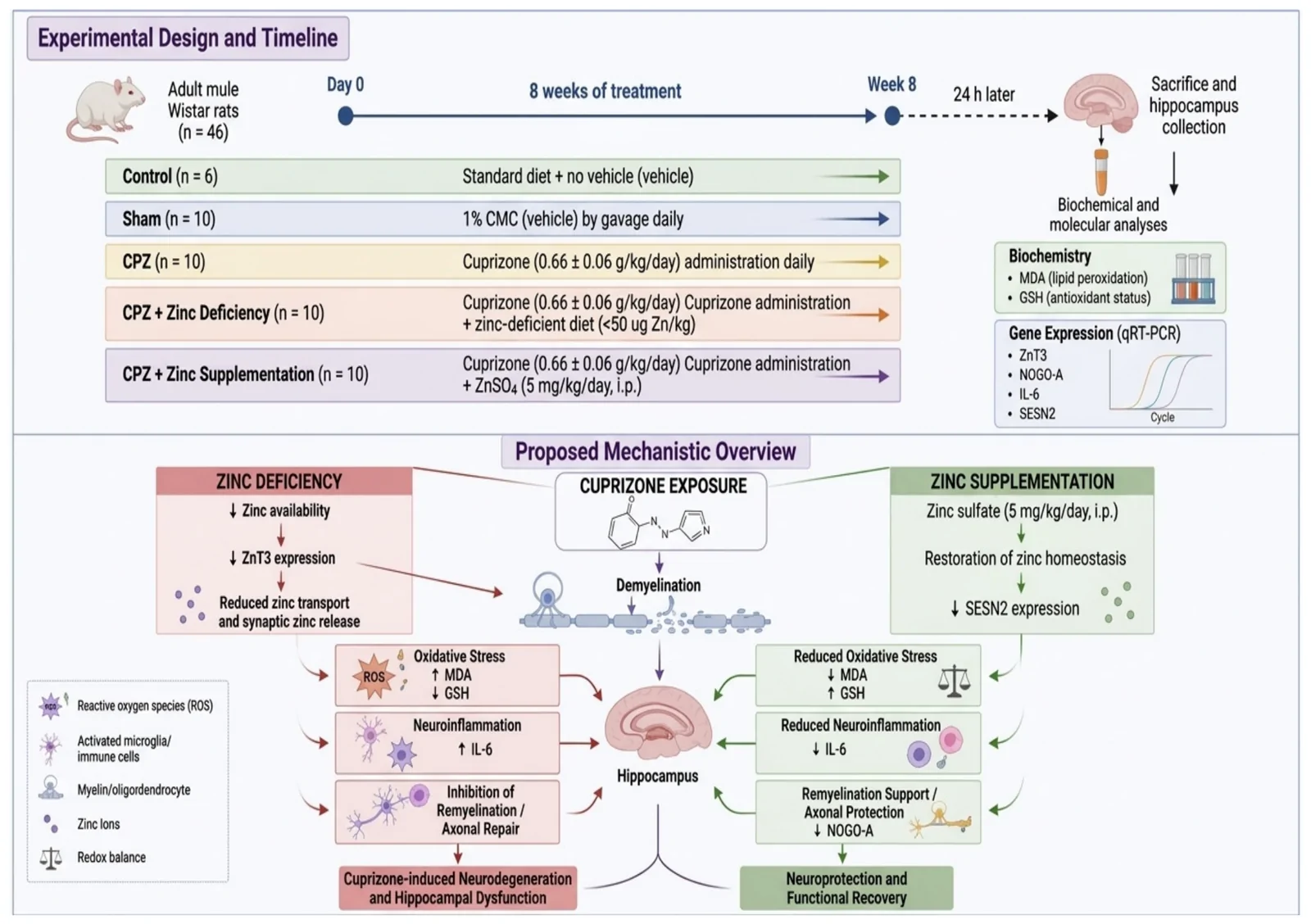
Experimental Design and Timeline.

The development of demyelination in the animals was evaluated using Rotarod tests and Myelin Basic Protein (MBP) gene expression analysis (described in detail in our published article, which is part of this study) [3], (Figure 4).

### 4.3. Tissue Sample Collection

At the end of the eight-week application period, rats were sacrificed under anesthesia with ketamine (75 mg/kg, i.p.; Ketalar, Parke-Davis, Detroit, MI, USA) and xylazine (10 mg/kg, i.p.; Rompun, Bayer, Leverkusen, Germany). Brain tissues were rapidly removed; hippocampal regions were isolated and stored at −80 °C until analysis.

### 4.4. Determination of Malondialdehyde (MDA) Levels

Lipid peroxidation levels in hippocampal tissues were determined using the thiobarbituric acid reagents (TBARS) method described by Uchiyama and Mihara. After homogenizing the tissue samples, appropriate chemical treatments were applied, and absorbance values were measured spectrophotometrically at 532 nm [55]. Results were expressed as nmol/g tissue.

### 4.5. Determination of Glutathione (GSH) Levels

Total glutathione levels in hippocampal tissues were evaluated using the Ellman method. Supernatants obtained from homogenized samples were incubated with the necessary reagents and absorbance values were measured at 412 nm [56]. Results were calculated as mg/g tissue.

All absorbance measurements for MDA and GSH were performed on a SPECTROstar microplate reader (BMG Labtech, Ortenberg, Germany).

### 4.6. RNA Isolation

Total RNA was isolated from hippocampal tissue using a commercial kit (Bio Basic, Markham, ON, Canada). Briefly, 25–50 mg of tissue was homogenized in lysis buffer on ice using a homogenizer (SONOPULS mini20, BANDELIN, Berlin, Germany), and the subsequent steps were performed according to the manufacturer’s instructions. RNA concentration and purity were assessed spectrophotometrically (SMA 1000, Merinton, Pekin, China), and samples with an A260/A280 ratio ≥ 1.9 were used for further analyses.

### 4.7. cDNA Synthesis

cDNA was synthesized from total RNA using a commercial reverse transcription kit (OneScript Plus, ABM, Vancouver, BC, Canada) according to the manufacturer’s instructions. An equal amount of total RNA (2 µg) was used for each sample, and the reactions were performed using a thermal cycler (Bio-Rad, Hercules, CA, USA). The concentration and purity of the resulting cDNA were assessed using a NanoDrop spectrophotometer (SMA 1000, Merinton, Pekin, China), and samples with an A260/A280 ratio of approximately 1.8 were used for subsequent analyses.

### 4.8. Real-Time PCR Analysis

The expression levels of ZnT3, NOGO-A, IL-6, and SESN2 were analyzed using a CFX96 Touch™ Real-Time PCR Detection System (Bio-Rad, Hercules, CA, USA). The primer sequences for the target genes and the reference gene, GAPDH, are provided in Table 5. qPCR reactions were performed using FastStart Essential DNA Green Master (Roche, Basel, Switzerland) in a total reaction volume of 20 µL. Thermal cycling conditions consisted of an initial denaturation at 95 °C for 10 min, followed by 45 cycles of denaturation at 95 °C for 10 s, annealing at 60 °C for 10 s, and extension at 70 °C for 10 s. Melt curve analysis was performed at 95 °C for 15 s, 60 °C for 10 s, and 95 °C for 10 s (Table 4). Relative gene expression levels were calculated using the 2^−ΔΔCt^ method [57].

### 4.9. Determination of Serum Zinc Concentrations

Two-milliliter blood samples collected from the animals were centrifuged at 3000 rpm for 10 min, and the resulting serum samples were immediately stored at −80 °C until further processing. Prior to analysis, the serum samples were diluted 1:50 in a 1% (*v*/*v*) Triton X-100 solution. Subsequently, serum zinc levels were measured using an inductively coupled plasma atomic emission spectrometer (ICP-AES; Varian Australia Pty Ltd., Mulgrave, Victoria, Australia). Data were expressed in µg/dL.

### 4.10. Statistical Analysis

Statistical analyses were performed using SPSS 26.0 (IBM, Armonk, NY, USA) software. Data are presented as mean ± standard deviation (SD). The normality of the data distribution was evaluated using the Shapiro–Wilk test. Differences between groups were determined using one-way analysis of variance (ANOVA), and post hoc comparisons were made using the Duncan test. A *p* < 0.05 value was considered statistically significant.

## 5. Conclusions

This study demonstrates that dietary zinc levels significantly influence hippocampal oxidative stress and molecular responses in a cuprizone-induced toxic demyelination model. Cuprizone-induced toxic demyelination was associated with lipid peroxidation (manifested by elevated MDA levels); GSH depletion; suppression of ZnT3 expression; and upregulation of NOGO-A, IL-6, and SESN2 gene expression.

Zinc deficiency exacerbated these demyelination-associated changes, suggesting that insufficient zinc levels may increase the hippocampus’s susceptibility to oxidative stress, neuroinflammation, disrupted zinc homeostasis, and alterations in pathways related to neuroplasticity. Conversely, zinc supplementation reduced MDA levels; restored GSH concentrations; increased ZnT3 expression; and suppressed the demyelination-associated upregulation of NOGO-A, IL-6, and SESN2.

Collectively, these findings indicate that zinc homeostasis may contribute to the molecular mechanisms underlying hippocampal damage in experimental demyelination. Therefore, zinc supplementation may exert potential protective transcriptional and antioxidant effects against the oxidative and inflammatory responses associated with hippocampal damage during toxic demyelination. In conclusion, the findings from this preliminary study provide essential information regarding the impact of dietary zinc status on the expression of hippocampal ZnT3, NOGO-A, IL-6, and SESN2 in a cuprizone-induced toxic demyelination model, and they pave the way for further studies that incorporate multiple time points.

### Limitations

This study has several important limitations that should be acknowledged.

First, our findings are based on a cuprizone-induced toxic demyelination model rather than a comprehensive model of human Multiple Sclerosis (MS). While this model is highly valuable for investigating metabolic demyelination and oligodendrocyte loss, it does not fully replicate the complex autoimmune mechanisms, multifocal inflammatory lesions, or broad neuroanatomical network pathologies characteristic of human MS.

Second, regarding systemic and regional zinc status, direct quantitative measurements of hippocampal zinc concentrations were not performed in this experimental cohort due to tissue limitations following real-time PCR and biochemical analyses (though serum zinc levels were evaluated). Future investigations should incorporate direct tissue-level quantification (e.g., via ICP-MS or AAS).

Third, zinc supplementation was administered via daily intraperitoneal (i.p.) injections; consequently, different bioavailability and tissue distribution profiles may exist compared to oral administration, which aligns more closely with standard clinical practice. Furthermore, potential injection-related chronic stress over an 8-week period must be taken into account.

Fourth, the evaluation of target molecular markers (ZnT3, NOGO-A, IL-6, and SESN2) was conducted solely at the transcriptional mRNA level via qRT-PCR. Because post-transcriptional and translational regulatory mechanisms can influence final protein abundance, subsequent studies incorporating Western Blot, ELISA, or immunohistochemical validations are warranted to confirm protein translation and spatial localization within hippocampal subfields.

Fifth, specific mechanistic pathways and inflammatory mediators—such as copper–zinc superoxide dismutase (Cu/Zn SOD) enzymatic kinetics, histopathological assessments of oligodendrocyte/myelin integrity, and broader microglial-derived pro-inflammatory cytokines (e.g., IL-1beta and TNF-alpha)—were not explored in this experimental setup.

Finally, our study evaluated hippocampal parameters at a single time point corresponding to active cuprizone-induced toxic demyelination, without analyzing the subsequent spontaneous remyelination and recovery phase following cuprizone withdrawal. Multi-phase temporal analyses and histological myelin assessments are required in future studies to clarify the precise role of zinc across both demyelination and remyelination stages.

Finally, as the current study is structured as a preliminary investigation based on a single experimental design, future research should incorporate additional time points and more comprehensive follow-up groups to fully elucidate the temporal dynamics.

Despite these limitations, our study provides valuable preliminary evidence regarding the regulatory role of zinc homeostasis on hippocampal oxidative balance and transcriptional responses during toxic demyelination.

## Figures and Tables

**Table 1:** Effects of Cuprizone-Induced Toxic Demyelination Model and Zinc Status on Hippocampal MDA and GSH Levels across Experimental Groups.

Groups	*n*	Hippocampus MDA (nmol/gr/Tissue)	Hippocampus GSH (mg/gr/Tissue)
Group 1—Control	6	256.17 ± 8.20 ^y^	268.74 ± 12.57 ^a^
Group 2—Sham	10	252.81 ± 26.06 ^y^	269.47 ± 26.71 ^a^
Group 3—CPZ	10	366.16 ± 54.19 ^x^	163.75 ± 22.23 ^c^
Group 4—CPZ + Zn deficient diet	10	395.82 ± 17.31 ^x^	163.71 ± 10.99 ^c^
Group 5—CPZ +Zn supplementation diet	10	275.03 ± 20.64 ^y^	244.81 ± 17.64 ^b^

Note. Data are expressed as mean ± standard deviation. Means sharing different lowercase superscript letters (x, y; a, b, c) within the same column are significantly different between groups (*p* < 0.05). Abbreviations: MDA, malondialdehyde; GSH, reduced glutathione; CPZ, Cuprizone-induced toxic demyelination; Zn, zinc.

**Table 2:** Effects of Cuprizone-Induced Toxic Demyelination Model and Zinc Status on Hippocampal NOGO-A and ZnT3 Relative mRNA Expression Levels.

Groups	*n*	Hippocampus NOGO-A (2^−ΔΔCt^)	Hippocampus ZnT3 (2^−ΔΔCt^)
Group 1—Control	6	0.82 ± 0.11 ^z^	1.21 ± 0.10 ^a^
Group 2—Sham	10	0.88 ± 0.10 ^z^	1.19 ± 0.09 ^a^
Group 3— CPZ	10	1.40 ± 0.19 ^y^	0.69 ± 0.14 ^b^
Group 4—CPZ + Zn deficient diet	10	1.97 ± 0.25 ^x^	0.49 ± 0.05 ^c^
Group 5—CPZ +Zn supplementation diet	10	0.89 ± 0.15 ^z^	1.24 ± 0.11 ^a^

Note. Data are expressed as mean ± standard deviation. Relative fold changes in mRNA gene expression were calculated using the 2^−ΔΔCt^ method. Means sharing different lowercase superscript letters (x, y, z; a, b, c) within the same column are significantly different between groups (*p* < 0.05). Abbreviations: NOGO-A, neurite outgrowth inhibitor A; ZnT3, zinc transporter 3; CPZ, CPZ, Cuprizone-induced toxic demyelination; Zn, zinc.

**Table 3:** Effects of Cuprizone-Induced Toxic Demyelination Model and Zinc Status on Hippocampal IL-6 and SESN2 Relative mRNA Expression Levels.

Groups	*n*	Hippocampus IL-6 (2^−ΔΔCt^)	Hippocampus SESN2 (2^−ΔΔCt^)
Group 1—Control	6	1.00 ± 0.09 ^z^	0.92 ± 0.05 ^d^
Group 2—Sham	10	0.99 ± 0.09 ^z^	0.89 ± 0.07 ^d^
Group 3—CPZ	10	1.92 ± 0.11 ^x^	1.51 ± 0.09 ^b^
Group 4—CPZ + Zn deficient diet	10	2.06 ± 0.14 ^x^	1.60 ± 0.06 ^a^
Group 5—CPZ +Zn supplementation diet	10	1.39 ± 0.39 ^y^	1.20 ± 0.06 ^c^

Note. Data are expressed as mean ± standard deviation. Relative fold changes in mRNA gene expression were calculated using the 2^−ΔΔCt^ method. Means sharing different lowercase superscript letters (x, y, z; a, b, c, d) within the same column are significantly different between groups (*p* < 0.05). Abbreviations: IL-6, interleukin 6; SESN2, sestrin 2; CPZ, Cuprizone-induced toxic demyelination; Zn, zinc.

**Table 4:** Serum Zinc Levels of the Study Groups (µg/dL).

Groups	*n*	Serum Zinc (µg/dL)
Group 1—Control	6	81.34 ± 6.35 ^b^
Group 2—Sham	10	82.49 ± 7.23 ^b^
Group 3—CPZ	10	51.06 ± 2.15 ^c^
Group 4—CPZ + Zn deficient diet	10	46.81 ± 4.89 ^d^
Group 5—CPZ +Zn supplementation diet	10	129.40 ± 6.10 ^a^

Data are expressed as means ± standard deviation (SD). Means sharing different lowercase superscript letters (a, b, c, d) within the same column differ significantly between groups (*p* < 0.001). Abbreviations: CPZ, Cuprizone-induced toxic demyelination; Zn, zinc.

**Table 5:** Primers used for real-time PCR.

Gene	Primer Sequence
ZnT3 Forward	5′-ACCACCTTCCTCTTCTCTATCT-3′
ZnT3 Reverse	5′-TCCCTTACAGGCTCAAATTCC-3′
NOGO-A Forward	5′-TCAAAGGTGACTGAGGCAGC-3′
NOGO-A Reverse	5′-ACTGGGCTGCACTACAGA-3′
IL-6 Forward	5′-GGGCTGCGATGGAGTCAGAG-3′
IL-6 Reverse	5′-TCCCTCACACAGGGCTCGAC-3′
SESN2 Forward	5′-TAGCCTACAGCCTCACCTATAA-3′
SESN2 Reverse	5′-CCAAAGACGCAGTGGATGTA-3′
GAPDH Forward	5′-GGGCCAAAAGGGTCATCATC-3″
GAPDH Reverse	5′-AACCTGGTCCTCAGTGTAGC-3′

## Data Availability

The datasets generated during and/or analysed during the current study are available from the corresponding author on reasonable request.
